# The Role of BDNF on Neural Plasticity in Depression

**DOI:** 10.3389/fncel.2020.00082

**Published:** 2020-04-15

**Authors:** Tao Yang, Zheng Nie, Haifeng Shu, Yongqin Kuang, Xin Chen, Jingmin Cheng, Sixun Yu, Huiying Liu

**Affiliations:** ^1^Department of Neurosurgery, The General Hospital of Western Theater Command, Chengdu, China; ^2^Department of Anatomy and Histology and Embryology, Regeneration Key Lab of Sichuan Province, Chengdu Medical College, Chengdu, China; ^3^Department of Respiratory and Critical Care Diseases, The Fifth Medical Center of PLA General Hospital, Beijing, China

**Keywords:** neurotrophic factors, BDNF, neural plasticity, neurogenesis, depression

## Abstract

Using behavioral, pharmacological, and molecular methods, lots of studies reveal that depression is closely related to the abnormal neural plasticity processes occurring in the prefrontal cortex and limbic system such as the hippocampus and amygdala. Meanwhile, functions of the brain-derived neurotrophic factor (BDNF) and the other neurotrophins in the pathogenesis of depression are well known. The maladaptive neuroplastic in depression may be related to alterations in the levels of neurotrophic factors, which play a central role in plasticity. Enhancement of neurotrophic factors signaling has great potential in therapy for depression. This review highlights the relevance of neurotrophic factors mediated neural plasticity and pathophysiology of depression. These studies reviewed here may suggest new possible targets for antidepressant drugs such as neurotrophins, their receptors, and relevant signaling pathways, and agents facilitating the activation of gene expression and increasing the transcription of neurotrophic factors in the brain.

## Introduction

Depression has been one of the major causes of mortality and morbidity in this century. It is the most common mood disorder occurs in all population ignoring social backgrounds. Depression can be classified as major depressive disorder (clinical depression), persistent depressive disorder (also called dysthymia), postpartum depression, psychotic depression, seasonal affective disorder, bipolar disorder, and so on. Although psychopharmacological agents, psychotherapy, and other options can be chosen for the treatment of depression, complete recovery can not be achieved in about 20–30% of patients treated with common antidepressants (Hirschfeld, 2012). To date, our knowledge about pathogenetic mechanisms of depression is limited (Racagni and Popoli, 2008; Jeon and Kim, 2016).

Several theories of depression have been proposed, including monoamine hypothesis, neuroendocrine mechanisms, neuroimmune and cytokine hypothesis. However, these theories have not been sufficient for completely explaining the pathology and treatment of depression. Recently, neural plasticity theories of depression have been widely developed. The neuroplasticity hypothesis is proposed. According to this hypothesis, the dysfunction of neural plasticity is an essential mechanism of depression (Kuhn et al., 2014; Liu et al., 2017). However, there is no authoritative research showing how neuroplasticity influences the disorder. Meanwhile, insufficient signaling by neurotrophic factors, an important role in neural plasticity, has been considered as a latent factor for depression and antidepressant responses have been observed while neurotrophin signaling promoted (Martinowich et al., 2007; Castrén and Rantamäki, 2010a,b; Autry and Monteggia, 2012; Castrén and Kojima, 2017).

Neurotrophic factors are compounds can bind to common tyrosine kinase receptors. Classic neurotrophic factors are a brain-derived neurotrophic factor (BDNF), nerve growth factor (NGF), neurotrophin-3 (NT-3) and neurotrophin-4 (NT-4; Bothwell, 2014). BDNF is the most representative neurotrophin linking to depression, while some studies report other neurotrophins linking to mood disorders (Castrén, 2014).

In this study, we review recent literature related to neural plasticity and the neurotrophic theories about depression and discuss how neurotrophins, specifically BDNF, play its role in depression through neural plasticity way.

## The Neurotrophic Factors and Neural Plasticity

The first neurotrophic factor, NGF, was discovered in the 1950s. BDNF was discovered in the 1980s and these two compounds established the neurotrophin family then (Cohen et al., 1954; Barde et al., 1982). The other two neurotrophins NT3 and NT4, were discovered since then (Lewin and Barde, 1996).

Releasing of neurotrophins is activated by neuronal activities (Thoenen, 1991). They fulfill the functions of neuronal survival and differentiation, synaptogenesis, and they regulate plasticity in an activity-dependent way (Park and Poo, 2013).

Physiological responses to neurotrophins are mediated by their binding to members of the tyrosine kinase receptor family (Trk) and the p75 neurotrophin receptor (p75^NTR^). NGF binds to TrkA, BDNF and NT4 bind to TrkB, and NT3 binds to TrkC (Reichardt, 2006; Bothwell, 2014). In contrast with Trk receptors, all neurotrophins can bind to p75^NTR^ and activate its downstream responses. p75^NTR^ binds both forms, mature and the uncleaved, of neurotrophins (Lee et al., 2001; Teng et al., 2005). When tyrosine kinase receptors are bound with neurotrophins, ligand-receptor dimerization and autophosphorylation will be induced in its intracellular tyrosine residues. Consequently, in the juxtamembrane region and the carboxyl terminus of the receptor, the phosphorylation of tyrosine residues (Tyr 515 and Tyr 816) evoked. Phosphorylation of these two tyrosine residues induces the interaction of the receptor with Shc (Src homology 2-containing protein) and phospholipase Cγ (PLCγ), respectively (Reichardt, 2006). The Ras–MAPK pathway, PI3K–Akt pathway, and the PLCγ–Ca^2+^ pathway are the three main downstream signaling cascades of activated Trk receptors (Kaplan and Miller, 2000; Leal et al., 2017).

### BDNF and Synaptic Plasticity

BDNF was originally thought to be an important regulator of early neuron development and survival (Barde et al., 1982), and it has recently been demonstrated in several processes in the mature brain, such as synaptic plasticity (Park and Poo, 2013). Early studies demonstrate that BDNF mRNA is upregulated by stimulation paradigm that induces long-term potentiation (LTP) in the hippocampus proper (Cornu Ammonis, CA, USA) region (Patterson et al., 1992). Mice with a target disruption on BDNF exhibit a significant impairment of LTP at Schaffer collateral-CA1 synapses (Korte et al., 1995). BDNF is required for late LTP in hippocampal CA1 synapses (Korte et al., 1998). TrkB mediates hippocampal LTP *via* recruitment of PLCγ and subsequent induction of cAMP response element-binding protein (CREB) and CaMKIV (Ca^2+^ and calmodulin-dependent protein kinase IV) phosphorylation (Minichiello et al., 2002). However, BDNF–TrkB signaling critical periods in LTP varies across synapses and according to the stimulation paradigm (Panja and Bramham, 2014). Meanwhile, LTP consolidation requires hours of BDNF–TrkB signaling at synapses of the dentate gyrus *in vivo* (Panja et al., 2014). In another research, LTP at CA3–CA1 hippocampal synapses were impaired in forebrain TrkB knockout mice (Minichiello et al., 1999). These findings corroborate the BDNF effects on synaptic LTP which require mitogen-activated protein kinase (MAPK) extracellular signal-regulated protein kinase (ERK) activation (Ying et al., 2002). Further investigation about the synaptic release of BDNF and its function during synaptic activation is needed to date.

Historically, the presynaptic effects of BDNF have been greatly attributed to modulation of the efficiency of vesicular glutamate release in mammalian synapses, including within the hippocampus (Tyler et al., 2002). Postsynaptic functions of BDNF in synaptic plasticity are that BDNF modulates the glutamate receptors. BDNF increases the trafficking and synaptic delivery of α-amino-3-hydroxy-5-methyl-4-isoxazole propionic acid (AMPA) receptors in hippocampal slices and hippocampal neuronal cultures (Caldeira et al., 2007; Fortin et al., 2012). The expression of a BDNF-dependent postsynaptic form of LTP was marked by increased insertion of AMPA receptors (Edelmann et al., 2015). Increasing the spine density of CA1 pyramidal neurons *via* MAPK/ERK pathway and Ca^2+^ entry *via* transient receptor potential canonical (TRPC) subfamily channel 3 are also related to exogenous application of BDNF (Alonso et al., 2004; Amaral and Pozzo-Miller, 2007). Several different studies reported the role of endogenous BDNF. Rex et al. (2007) demonstrated that actin polymerization *via* BDNF–TrkB signaling-mediated recruitment of p21-activated kinase (PAK)-cofilin pathways is needed in TBS-induced LTP using TrkB-Fc chimera. Meanwhile, Lai et al. (2012) reported BDNF- and glutamate-induced spine head enlargements are needed by activation of the Rho-GTPase Rac1 *via* a direct interaction of phosphorylated TrkB and the guanine exchange factor Tiam1. In another study, spike timing-induced spine enlargements require endogenous BDNF and protein synthesis at CA1 neurons (Tanaka et al., 2008).

According to the evidence previously described, exogenous BDNF has been shown to promote LTP-induced spine enlargement and activity-induced increase in synaptic Homer2b levels (Briz et al., 2015). The effect of BDNF on structural plasticity may also depend on the local synthesis of cytoskeleton related proteins (such as RhoA and LIMK1) induced by this neurotrophic protein (Bosch et al., 2014). The application of BDNF and the developmental stage of neurons directly affects the role of BDNF (Ji et al., 2010) to read a full description of BDNF-mediated structural changes at synapses (Zagrebelsky and Korte, 2014). Synaptic activity has been shown to regulate synaptic structure by regulating the dendritic synthesis of BDNF, which may help to further enhance the local effect of neurotrophic factors (Verpelli et al., 2010).

Besides this BDNF-mediated modification of mature synapses, BDNF plays a crucial role in neural development and neurogenesis. BDNF has been shown to stimulate the proliferation of NPCs and promote long-term survival of their progeny (Katoh-Semba et al., 2002; Sairanen et al., 2005; Scharfman et al., 2005). Both intrahippocampal infusion of BDNF and its peripheral injection have been associated with potent stimulation of hippocampal neurogenesis (Shirayama et al., 2002; Scharfman et al., 2005; Schmidt and Duman, 2010). Kuipers et al. (2016) reported that promoted neurogenesis is associated with BDNF-LTP in the DG. Inhibition of Arc translation blocks BDNF-LTP induction and the associated pro neurogenic effects. Interestingly, basal rates of proliferation and newborn cell survival are unaltered in Arc knockout mice. While the mechanisms remain to be explored, these findings link the pro neurogenic effects of acute BDNF infusion to the induction of Arc dependent LTP in the adult rodent DG (Leal et al., 2017).

### Pro-forms of Neurotrophins

BDNF and other neurotrophins are first synthesized as a form of precursor protein and processed into a mature form by proteases (Seidah et al., 1996). The proneurotrophins proNGF, proBDNF and proNT3 bind a complex composed of either sortilin or SorCS2 (two members of the VPS10 family) and p75^NTR^ (Lee et al., 2001; Hempstead, 2014). The activation of this receptor complex will initiate cell death and it is the essential procedure of proneurotrophins (Nykjaer et al., 2004; Deinhardt et al., 2011). Subsequently, activation of Trk receptors promotes neuronal survival, neuroplasticity, and synaptogenesis, meanwhile, p75^NTR^ enhances cell death and synaptic pruning (Deinhardt and Chao, 2014; Kraemer et al., 2014). Studies revealed that BDNF-dependent long-term depression (LTD) occurs when proBDNF processed into BDNF, and hippocampal LTD depends on the activation of p75^NTR^ activation (Pang et al., 2004; Woo et al., 2005).

**Figure 1:**
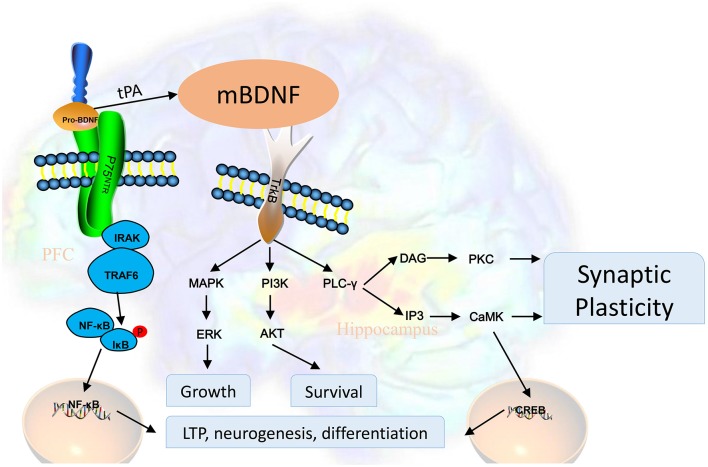
BDNF is first synthesized as proBDNF and processed into BDNF. BDNF activates tyrosine kinase receptors and subsequently promotes neuronal survival, neuroplasticity, and synaptogenesis through different signaling pathways. Activated Trk receptors recruit tyrosine kinase substrates, including PI3K and PLC-γ. PLC-γ binds to Tyr-790 and this interaction has been proposed to facilitate interactions with ion channels, such as the VR1 capsaicin channel. Through residue Tyr-490, Shc or FRS2 become tyrosine phosphorylated and provide a scaffold for other signaling proteins that lead to the activation of the Ras/MAPK (mitogen-activated protein kinase) or the PI3K/Akt pathways. Abbreviations: AKT, protein kinase B; BDNF, brain-derived neurotrophic factor; CaMK, calmodulin-dependent protein kinase; PKC, protein kinase C; CREB, cAMP response element-binding protein; ERK, extracellular signal-regulated kinases; IκB, nuclear factor of kappa light polypeptide gene enhancer in B-cells inhibitor; IP3, inositol trisphosphate 3; IRAK, interleukin-1 receptor-associated kinase; MAPK, mitogen-activated protein kinase; NF-κB, nuclear factor kappa-light-chain-enhancer of activated B cells; p75NTR, p75 neurotrophin receptor; PI3K, phosphoinositide 3 kinase; PLC-γ, phospholipase C gamma; tPA, tissue plasminogen activator; TRAF6, tumor necrosis factor receptor-associated factor 6; Trk, activates tyrosine kinase.

The BDNF pro-domain acts as a ligand according to several recent reports. Large dense-core vesicles could be found in excitatory presynaptic terminals of the adult mouse hippocampus, these large dense-core vesicles are BDNF and its pro-peptide (Dieni et al., 2012). The BDNF pro-peptide can promote hippocampal LTD (Mizui et al., 2015). It is also demonstrated that LTD was completely inhibited in hippocampal slices treated with the BDNF pro-peptide with the Met mutation (Mizui et al., 2015). Acute growth cone retraction and a decrease were induced by application of the BDNF pro-peptide with the Met mutation in Rac activity in hippocampal neurons (Anastasia et al., 2013). According to these findings, a post-translational mechanism of BDNF, proteolytic cleavage of proBDNF could be new mechanisms for the development of brain diseases (Castrén and Kojima, 2017). Trk receptor signaling is initiated by dimerization and autophosphorylation at specific tyrosine residues. After binding, activated Trk receptors recruit adaptor proteins such as Shc and FRS2 and other important tyrosine kinase substrates, including phosphoinositide 3-kinase (PI3K) and phospholipase C-γ (PLC-γ). The key docking sites on Trk receptors are Tyr-490 (Tyr-496 in human TrkA) in the juxtamembrane region and Tyr-790 (Tyr-791 human TrkA) in the tail of the cytoplasmic domain. PLC-γ binds to Tyr-790 and this interaction has been proposed to facilitate interactions with ion channels, such as the VR1 capsaicin channel. Through residue Tyr-490, Shc or FRS2 become tyrosine phosphorylated and provide a scaffold for other signaling proteins that lead to the activation of the Ras/MAPK or the PI3K/Akt pathways. These phosphorylation events have many consequences. The signaling pathway of BDNF is summarized in Figure 1.

## Neuroplasticity Dysfunction in Depression

Neural plasticity can be defined as a neuronal adaptation that is an individual response to the environment, which includes new cell formation and genetically healthy cell death in the adult brain (Duman et al., 1999). Mechanism of depression and the antidepressant action can be explained using the concept of an intracellular signal transduction cascade and neural plasticity. Each specific neural circuit is activated by learning, memory, stress, or the environment, which induce an intracellular signal transduction cascade that is a core function of neural plasticity. The signal transduction cascade plays a key role in regulating neuronal atrophy, neuronal death, and neurogenesis (Jeon and Kim, 2016).

### Neurogenesis and Depression

The most commonly studied brain region in neurogenesis research is the hippocampus. Several factors have been demonstrated to regulate hippocampal neurogenesis, including exercise (Erickson et al., 2011), hormones (Dranovsky and Hen, 2006) and environment (hippocampus-dependent learning, Anderson et al., 2011) suggesting that neurogenesis is closely related to some physiological mechanisms. Some negative stress and adverse experiences can lead to a significant decrease in the proliferation of granulosa cells, thereby affecting the function of the brain and hippocampus, among which the effect on memory is of great concern (Duman et al., 1999; Colla et al., 2007), while chronic antidepressant treatment can reverse this effect (Duman et al., 2001). Studies show reduced hippocampal volume in depressed patients (MacQueen et al., 2003). Meanwhile, in animal models, Reduction in hippocampal volume and a decrease of neurogenesis can be observed (Banasr et al., 2004). Although it is debatable whether hippocampal shrinkage is a result of depression or a preexisting vulnerability marker for depression, it does suggest that structural changes in the brain may be closely related to environmental factors, genetic risk, and outcome. Patients with the short (S) variant tri-allelic polymorphisms of the serotonin transporter gene (5-HTTLPR) promoter region were more likely than those with only one risk factor (genetic or environmental) to have smaller hippocampal volumes when experience childhood stress (Frodl et al., 2010).

### Synaptic Plasticity and Depression

Stressed rodents display abnormal patterns of synaptic plasticity in brain areas including the hippocampus and prefrontal cortex (Krishnan and Nestler, 2008). N-methyl-d-aspartic acid (NMDA) receptor antagonist ketamine has a long-lasting antidepressant effect and can reverse depressive symptoms (Berman et al., 2000; Zarate et al., 2006) by improving the abnormal plasticity of glutamate synapses (Duman and Aghajanian, 2012). Repeated stress lowers the dendritic complexity of the prefrontal cortex and hippocampal neurons (Radley et al., 2006a,b) and selective deletion of excitatory synaptic markers (Tzanoulinou et al., 2014; Kaster et al., 2016). As with stressed rodents, synaptic markers in the frontal limbic region changed in MDD patients (Feyissa et al., 2009; Duric et al., 2013). These data support the hypothesis that depression is caused by abnormal synaptic plasticity in the affected area (Duman and Aghajanian, 2012). Using the developing visual cortex, studies had shown that long-term use of the antidepressant fluoxetine can reactivate the plasticity of the adult cortex, which is difficult to distinguish from the plastic enhancement usually found in the juvenile cortex (Maya Vetencourt et al., 2008). When this promoted state of plasticity is combined with rehabilitation, plastic networks can reorganize so that impaired vision of one eye, due to developmental visual deprivation, can be fully restored (Castrén and Kojima, 2017).

Plenty of evidence showed a significant reduction in hippocampal volume in depression patients (Chan et al., 2016). These changes may result from a neurodegenerative reaction to increased glucocorticoid levels in depression (Sheline, 2011). Alcaide et al. (2019) reported alterations of perineuronal nets in the prefrontal cortex of patients with bipolar disorder. The underlying mechanism is that alterations of Perineuronal nets reduced neuronal plasticity of the prefrontal cortex and thus affected the patients.

## The Neurotrophic Factors and Depression

Many studies have demonstrated that BDNF plays an important role in the pathophysiology of several psychiatric disorders and the mechanism of action of psychotropic drugs (Molteni et al., 2009b; Calabrese et al., 2011; Björkholm and Monteggia, 2016). These data provide direct evidence to support the neurotrophic hypothesis of depression, which has been shown to decrease the expression of neurotrophic factors in depressed patients and can be reversed with effective antidepressant treatment.

### BDNF in Depressed Patients and Animal Model

Guilloux et al. (2012) found that the expression of BDNF and its receptor TrkB was decreased in postmortem brain samples of depressed patients (Guilloux et al., 2012; Thompson, 2012; Tripp et al., 2012). Similarly, in the brain samples of depressed patients, the expression of BDNF in the hippocampus of subjects who took antidepressants was higher than that of subjects who did not take antidepressants (Chen et al., 2001). The expression of BDNF was negatively correlated with the severity of depression, and female patients had lower BDNF levels (Sen et al., 2008). Multiple reports and meta-analyses have shown that patients with depression have lower blood BDNF levels (Karege et al., 2002; Bocchio-Chiavetto et al., 2010; Molendijk et al., 2011, 2014). It is not known whether the decrease of serum BDNF level is related to the decrease of BDNF level in the brain, peripheral tissues or both (Sen and Sanacora, 2008; Sen et al., 2008).

In animal models of stress, BDNF levels are reduced in both cortex and hippocampus (Duman and Monteggia, 2006; Molteni et al., 2009a). This reduction could also be found in social stress mice (Tsankova et al., 2006; Martinowich et al., 2007). Mice with loss of BDNF expression in a specific area of forebrain show behaviors like the depressed ones (Monteggia et al., 2007; Lindholm and Castrén, 2014). The knockdown of TrkB produces anxiety-like behavior (Bergami et al., 2008) and the deletion of BDNF increases depression-like behavior (Taliaz et al., 2010). Furthermore, TrkB interaction with glucocorticoid receptor signaling pathway in the cytoplasm and at the genome level was also investigated. Dexamethasone and BDNF-induced expression of a unique group of genes activated neuron growth and differentiation and dexamethasone (Lambert et al., 2013). Glucocorticoids may under certain conditions act as TrkB activators, and BDNF increases serine phosphorylation of the glucocorticoid receptor (Jeanneteau et al., 2008).

**Figure 2:**
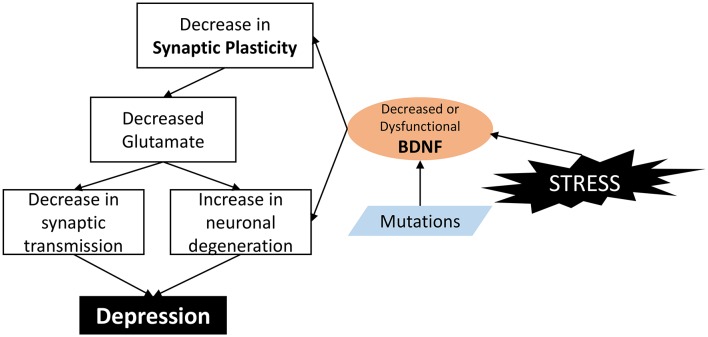
How alteration of functional BDNF result in depression. In brief, BDNF is a molecule involved in the control of synapse formation and regulation of activity-dependent changes in synapse structure and function. BDNF stimulation increases synaptic spine density by a mechanism dependent on the Ras/ERK pathway. Dysfunction or decreased BDNF leads to malfunction of synaptic plasticity, and decreased excitatory neurons and glutamate; and eventually lead to depression.

However, in animal models of stress, BDNF levels in the nucleus accumbens were increased (Krishnan and Nestler, 2008; Castrén and Kojima, 2017). Almost all the classes of antidepressants increase BDNF expression, while the behavioral effects of two different classes of antidepressants, i.e., fluoxetine and desipramine, are abolished in BDNF-deficient mice (Saarelainen et al., 2003). Different antidepressants seem to have different effects in healthy rats (Dwivedi et al., 2006). So BDNF may have different effects on depression-like behavior in the different brain areas and neuron networks (Castrén and Rantamäki, 2010a,b; Castrén and Kojima, 2017). Figure 2 showed a diagram about how alteration of functional BDNF results in depression.

### BDNF Alleles and Depression

Single nucleotide polymorphisms (SNPs) in the BDNF gene may be one of the important factors leading to brain dysfunction, which can lead to depression. The most typical SNP of Bdnf, the Val66Met (rs6265), replaces valine/methionine in the prodomain domain, thereby altering the sorting of proteins and its availability in the synaptic gap (Egan et al., 2003). Mice with the Met/Met or Val/Met alleles of BDNF had smaller hippocampal volumes, while those with the Met/Met gene had reduced dendritic complexity (Egan et al., 2003), and impaired working memory (Yu et al., 2012). Frodl et al. (2010) found a small hippocampal volume in depressed and healthy individuals with the BDNF Met allele. Interestingly, hippocampal function may be regulated differently by this polymorphism in patients and controls (Eisenberg et al., 2013). However, when we explored the role of Val66Met polymorphism in 152 depressed patients and 255 healthy controls, we found no significant difference in the allele frequency of BDNF polymorphism between affected and unaffected subjects (Niitsu et al., 2014). To better describe the sequence variability of the BDNF gene, in-depth sequencing was performed on 272 depressed patients and 264 control subjects. The results showed 83 new BDNF gene SNPs, six of which were related to depression (Eisenberg et al., 2013). It was found that subjects with the BDNF Met allele and 5-HTTLPR type were at increased risk of depression after experiencing stress (maltreatment; Taylor et al., 2007) these studies suggest that this combination of genes (intergene interactions) increases the risk of environmental events, thereby affecting the vulnerability/resilience balance.

Lee et al. (2001) reported that Val66Met mutation alters anxiety-related behavior in mice (Chen et al., 2006). Further researches revealed that in the hippocampus of mice with this polymorphism are defective in NMDAR-dependent plasticity (Ninan et al., 2010). Liu et al. (2012) reported that mice expressing the human BDNF val66met SNP have an attenuated antidepressant response following acute ketamine administration.

Several clinical studies are initiated following these preclinical findings linking BDNF to the antidepressant response to ketamine. Laje et al. (2012) proposed that alterations in BDNF function can impact ketamine’s antidepressant effects through a small trial investigated ketamine treatment outcomes in patients with major depression carrying either the functional BDNF val66val allele against met carriers found an increased antidepressant response to ketamine in individuals with the Val/Val than met carriers. Haile et al. (2014) found that low dose ketamine infusion increasing the serum concentration of BDNF, especially in responders to treatment suggesting plasma BDNF may be a potential biomarker for the antidepressant effects of ketamine. Nevertheless, paradoxical clinical results have also been reported (Machado-Vieira et al., 2009). The relationship between BDNF in the brain and peripheral levels of BDNF are unclear. However, given the critical need for biomarkers in the field of depression, the potential ability to correlate BDNF plasma levels with antidepressant responses to ketamine may warrant further investigation.

Other reported SNPs of BDNF were rs1048218, rs1048220, rs2049046, and rs11030094. In cultured hippocampal neurons, BDNF rs1048220 could inhibit the cleavage of pro-BDNF (Koshimizu et al., 2009). However, no studies available showed BDNF rs1048218 and rs1048220 associated with depression (Aldoghachi et al., 2019). Recently in a large pharmacogenetics study, Hennings et al. (2019) reported BDNF rs2049046 and rs11030094 might promote antidepressant treatment response in depressed patients.

### BDNF in the Antidepressant Responses

The role of neurotrophins in the mechanism of antidepressant treatments is much clearer than their role in depression (Lindholm and Castrén, 2014; Castrén and Kojima, 2017). In the 1990s, Nibuya et al. (1995, 1996) found that antidepressant drugs and electroconvulsive therapy could enhance BDNF and TrkB mRNA expression in the hippocampus and cortical regions in a timeframe similar to the onset of the antidepressant-like response. Subsequent work suggested that DG and CA3 may be key regions for antidepressant effects acting through BDNF. And the antidepressant effects may be mediated by TrkB activation of MAPK, acting through the ERK pathway (Shirayama et al., 2002). Another surprising noted in this work is that the antidepressant effect lasted up to 10 days after the BDNF infusion, well past the time frame of the degradation of BDNF, suggesting that neurotrophins may be triggering a sustained plasticity mechanism to mediate the long-term antidepressant effects (Shirayama et al., 2002). To examine a potential role for BDNF selectively in the brain in mediating antidepressant effects, studies have utilized inducible and conditional knockout mice in which the deletion of BDNF is controlled regionally and temporally. Monteggia et al. (2004) generated an inducible knockout mouse in which BDNF was selectively deleted in broad forebrain regions and these mice did not show alterations in depression-related behavior. However, these mice had an attenuated response to the antidepressant desipramine suggesting forebrain BDNF was required for antidepressant efficacy. Using an adeno-associated virus (AAV) expressing Cre recombinase or AAV expressing green fluorescent protein (GFP) as a control, BDNF was selectively deleted in subregions of the hippocampus in adult floxed BDNF mice. The loss of BDNF in either the DG or CA1 subregion of the hippocampus did not alter depression-related behavior (Adachi et al., 2008). These data suggested that BDNF in the hippocampus, specifically in particular subregions, may be critical for antidepressant responses in humans.

BDNF expression can be promoted by fluoxetine in the visual cortex and the hippocampus. The effect of antidepressant therapy is mediated by BDNF signal and dependent on BDNF signal (Maya Vetencourt et al., 2008). The regulation of hydroxytryptamine and neuro-suppression also requires the effect of fluoxetine on plasticity (Maya Vetencourt et al., 2011; Maya-Vetencourt and Origlia, 2012). However, it is unclear how these different systems interact to promote plasticity. Chronic fluoxetine treatment promotes the developmental plasticity of the dentate gyrus in the hippocampus (Hagihara et al., 2013). This phenomenon, known as “desaturation,” is characterized by increased expression of markers in immature dentate granule neurons, while mature markers decrease not only in newborn granule neurons, but also in older granule neurons (Hagihara et al., 2013).

The discovery that NMDA receptor antagonist ketamine can revert depressive symptoms with a long-lasting antidepressant effect, has generated a great deal of interest in unraveling the underlying biological mechanisms mediating the fast-acting response (Berman et al., 2000; Zarate et al., 2006). Another study showed the antidepressant-like effect of ketamine was attenuated in inducible forebrain specific BDNF and conditional TrkB knockout mice (Autry et al., 2011). Ketamine was shown to rapidly increase the phosphorylation of TrkB, an indicator of TrkB activation, in the hippocampus. This rapid increase in BDNF protein expression while required, was not maintained at 24 h after ketamine treatment, suggesting that BDNF working through TrkB was triggering intracellular signaling, and possibly synaptic plasticity effects, required for the antidepressant response (Autry et al., 2011). Other works also confirmed the importance of this pathway in the rapid antidepressant response to ketamine as eEF2K null knockout mice that are administered an acute low dose of ketamine do not have increased BDNF protein expression and do not show an antidepressant response to the drug (Nosyreva et al., 2013). Studies suggested that ketamine activates the mammalian target of rapamycin (mTOR) through disinhibition of glutamate transmission, resulting in a rapid burst of glutamate that triggers BDNF release to increase synapse formation (Duman, 2014). Previous work examining the antidepressant effects of ketamine demonstrated that mTOR was downstream of BDNF (Autry et al., 2011). It is quite possible that ketamine by blocking NMDA receptors at rest, inhibiting eEF2K and de-suppressing protein synthesis of BDNF, and other targets, may then be triggering activation of mTOR. Indeed, BDNF mice with the met/met SNP do not response to the antidepressant effects of ketamine and have impaired synaptogenesis that is believed to be mediated through mTOR (Liu et al., 2012) supporting the notion that BDNF is upstream of this signaling pathway. Recent work has shown that a 30-min treatment of slice preparations with ketamine potentiates synaptic efficacy in the Schaffer collateral pathway from CA3 to CA1 subregions of the hippocampus (Nosyreva et al., 2013, 2014). The ketamine triggered a rapid increase in BDNF protein expression was shown to rapidly increase surface expression of the AMPA receptor subunits, GluA1 and GluA2, in the hippocampus that was required for the increase in synaptic efficacy as well as the antidepressant effects of ketamine (Nosyreva et al., 2013). This ketamine-induced increase in synaptic efficacy was age-dependent as young mice did not exhibit this increase in synaptic potentiation or display an antidepressant response to ketamine (Nosyreva et al., 2014).

## What Beneath Neurotrophic Hypothesis Is Neural Plasticity

The above findings suggest that Impaired neuroplasticity indicates abnormal changes in neurogenesis, axon branching, dendrites, and synapses. Abnormalities in neuroplasticity may be related to changes in levels of neurotrophic factors, especially BDNF, which plays a key role in neuroplasticity. The synthesis and secretion of BDNF are activity-dependent, a phenomenon related to neuronal plasticity (Caldeira et al., 2007; Castrén and Rantamäki, 2010a; Chen et al., 2011; Bothwell, 2014; Briz et al., 2015). The Bdnf gene structure consists of nine exon 5′ translation, each link to each area, and a 3′ coding exons (IX; Aid et al., 2007). The structure of the Bdnf gene has nine exons 5′ translation, each link to each area, and 3′ coding exons (IX; Aid et al., 2007), and human Bdnf gene contains 11 exons and nine promoters, because of two human exons (Pruunsild et al., 2007). Among them, promoter IV is highly sensitive to neuronal activity, which is associated with a transient increase in [Ca^2+^] I (Patterson et al., 1992). BDNF transcription can occur at the synaptic level; Selective regulation of different subtypes may regulate the expression of neurotrophic proteins in a very specific way, providing a spatial encoding that facilitates intercellular communication. It was proposed neuronal plasticity and the antidepressant response might have a relationship since antidepressants were shown to promote BDNF expression and signaling (Castrén, 2014; Castrén and Kojima, 2017). Several activity-inducing paradigms, such as high-frequency stimulation (HFS; Patterson et al., 1992), strongly induce the expression of the BDNF mRNA. In animal models, stress reduces the long 3′ UTR Bdnf mRNA levels in the prefrontal cortex of adult rats (Luoni et al., 2014, 2016) and hippocampus of adolescent rats (Berry et al., 2015). Activity-dependent structural changes at synapses, for example, increased number and volume of dendritic spines are believed to support alterations in the levels of glutamate receptors and signaling molecules, and ultimately to sustain synaptic strength (Kasai et al., 2010). BDNF plays a critical role in this structural plasticity. Some *in vitro* studies support the view that in the hippocampus, BDNF increases the branches and growth of developing neurons, while regulating the spinal density and morphology of mature neurons (Ji et al., 2010; Zagrebelsky and Korte, 2014). Together, these results suggest that negative stimulation, like stress, reduces BDNF synthesis in the transcriptional level, and leads to impairment of structural neuroplasticity. As a result, these impairments of structural neuroplasticity may be the initial factor of depression.

A large number of studies corroborate the idea that neuronal plasticity would be promoted by neurotrophic factors and this promotes antidepressant responses in depressed patients correspondingly (Nestler et al., 2002; Castrén and Hen, 2013; Castrén and Kojima, 2017). Antidepressants modulate neuronal plasticity at several levels (Castrén and Hen, 2013): first, chronic antidepressant and acute ketamine treatment promotes synaptogenesis and synaptic strength. These effects of ketamine are BDNF-dependent according to the above discussions. Second, enhanced neurogenesis, which depends on BDNF signaling (Sairanen et al., 2005), can be detected by antidepressants in the dentate gyrus (Santarelli et al., 2003; Sahay and Hen, 2007). Finally, antidepressants increase axon elongation and dendritic sprouting (Chen et al., 2011) as well as the expression of plasticity-related proteins (Sairanen et al., 2007). Although many details remain to be investigated about whether mediated by BDNF or TrkB signaling, BDNF is known to influence both axonal and dendritic sprouting (Cohen-Cory et al., 2010). These data suggest that there is a strong correlation between neuroplasticity and antidepressant effect, and in some cases, there is causality, at least to some extent, the effect of plasticity is mediated by BDNF signal.

## Conclusions

A large number of studies showed neurotrophic factors, especially BDNF showed a close relationship with depression. Alterations of functional neurotrophic factors may result in the pathophysiology of depression by its mechanism of attenuating neural plasticity. Enhancement of neurotrophic factors signaling has great potential in therapy for depression. Although many details remain to be investigated, two potential directions can be inspired: molecules derived neurotrophins pathways might become a biomarker for depression. The neurotrophins initiated Trk signaling pathway may play an important role in screening or novel antidepressant drugs. There is no “unified theory” to perfectly explain the nature of depression. Mechanisms that promote depressive symptoms in response to stress differ markedly between different neural circuits. Researchers and clinicians must use a multidisciplinary approach to explore the neurobiological bases for depression’s many subtypes.

## Author Contributions

TY and ZN researched and wrote this review. SY and HL conceived the research. HS, YK, XC, and JC assisted in writing and editing.

## Conflict of Interest

The authors declare that the research was conducted in the absence of any commercial or financial relationships that could be construed as a potential conflict of interest.
